# Comparison of Bone-Patellar Tendon-Bone Graft, Semitendinosus–Gracilis Graft and Semitendinosus–Gracilis with Preserved Tibial Insertion Graft in Anterior Cruciate Ligament Reconstruction in Sports Persons

**DOI:** 10.5704/MOJ.2107.003

**Published:** 2021-07

**Authors:** A Soni, RK Gupta, M Raghav, GD Masih, P Bansal

**Affiliations:** Department of Orthopaedics, Government Medical College and Hospital, Chandigarh, India

**Keywords:** cruciate ligament, reconstruction, graft, semitendinosus, gracilis

## Abstract

**Introduction:**

Bone-patellar tendon-bone (BPTB) and semitendinosus–gracilis (STG) are the commonest grafts used for ACL reconstruction. However even after having been debated for years, there is no consensus about the ideal graft. Moreover, the literature is deficient about STG graft with preserved tibial insertion (STGPI) which preserves the proprioception. Our aim is to compare the outcome of BPTB, free STG and STGPI grafts after ACL reconstruction in professional sports persons. We compared the outcome in terms of mechanical stability, functional outcome, return to sports activity and degenerative changes.

**Material and Methods:**

Professional sports persons aged between 16-50 years operated for ACL tear using BPTB, free STG and STGPI grafts with minimum follow-up of two years were identified from hospital records. Patients with associated knee injuries were excluded. Patients, divided in three groups according to graft used, were compared in terms of mechanical stability (arthrometric examination KT-1000 score), functional outcome (Lysholm Score), return to sports activity (Tegner score and difference in thigh circumference) and degenerative changes (KL grading).

**Results:**

BPTB graft group was found to be better than free STG and STGPI graft groups in terms of KT-1000 score. There was no statistically significant difference among the groups in terms of Lysholm score, Tegner score, difference in thigh circumference and KL grading.

**Conclusion:**

BPTB graft is better than free STG and STGPI grafts in terms of knee stability. When compared for patient reported outcome, return to sports activity, osteoarthritic changes and graft failure there is no significant difference among the three types of grafts.

## Introduction

Anterior cruciate ligament (ACL) reconstruction is a commonly performed surgery for ACL deficient knee. The results of this surgery depend upon various factors including age and sex of patient, duration of injury, pre-operative knee function and type of graft^1,2^.

Allograft, as compared to autograft is considered to achieve poorer functional outcome which is not desirable in high demand patients like sports persons^3-5^. Among the autografts, though debated since years, controversy still exists regarding the superiority of bone-patellar tendon-bone (BPTB) vs semitendinosus–gracilis (STG) graft. However, there is no ideal graft for ACL reconstruction^1^.

Also, it has been reported that a proprioception deficient knee, because of ACL injury, cannot recover fully after reconstruction surgery^6^. Though the preservation of insertion of semitendinosus and gracilis tendons during graft harvesting for ACL reconstruction has been reported to preserve the proprioception of the knee joint^7^. There is a paucity in the literature about the functional outcome of this graft for ACL reconstruction as compared to other grafts.

Our study is a post-hoc analysis where we compared the mid to long term outcome of BPTB vs free STG vs semitendinosus–gracilis with preserved tibial insertion (STGPI) grafts after ACL reconstruction in professional athletes.

We compared the three types of grafts in terms of objective mechanical stability, functional outcome, return to sports activity, degenerative changes and graft failure.

## Materials and Methods

The present study is a post-hoc analysis of a retrospective cohort. The professional sports persons aged between 16-50 years operated at our institute from 2011-2016 for ACL tear using BPTB, free STG and STGPI grafts with a minimum follow-up of two years were identified from our hospital record. We excluded the patients with multi-ligament knee injuries / associated meniscus injuries. The patients were contacted on phone and called for follow-up in our outpatient department.

After calculating the sample size of 90, 102 patients divided in three groups were enrolled in the present study with 34 patients in each group. Group I for BPTB graft, Group II for free STG graft and Group III for STGPI graft. First 34 patients reported in each group were enrolled after taking written consent. All the patients were assessed for mechanical stability (arthrometric examination KT-1000 score), functional outcome (Lysholm Score) return to sports activity (Tegner score and difference in thigh circumference) and degenerative changes (KL grading).

All the patients were treated using standard techniques for ACL reconstruction. Our post-operative rehabilitation protocol was similar in all patients. Clearance from institutional ethical board was taken before starting the study.

ANOVA test was used to compare the three groups for KT-1000 score, Lysholm score, Tegner score and difference in thigh circumference. Post Hoc Tukey HSD test was used to compare Group I with Group II, Group I with Group III and Group II with Group III for KT-1000 score, Lysholm score, Tegner score and difference in thigh circumference.

## Results

A total of 102 patients were enrolled in this study. There were four graft failures; one in Group I, two in Group II and one in Group III. The difference was not significant among groups (ANOVA test). We analysed 98 patients at final follow-up with mean follow-up of 28.79, 33.79 and 29.54 months in Group I, Group II and Group III, respectively. Mean age in Group I, Group II and Group III was 24.0 +/5.0, 23.21 +/- 7.5 and 23.0 +/- 5.7 years, respectively. Three groups were compared and found to be matched for age (Table I). There were more males than females and right side was involved more frequently (Table II). Duration of injury before surgery ranged from three weeks to seven years (Table III).

**Table I:** Age distribution

	Age in years (Mean +/- S.D)	P value*
Group I (n=33)	24.0 +/- 5.0	0.888
Group II (n=32)	23.21 +/- 7.5	
Group III (n=33)	23.0 +/- 5.7	

**Table II:** Sex distribution and side of involvement

	Male		Female	
	Left	Right	Left	Right
Group I (n=33)	18	14	1	0
Group II (n=32)	14	17	1	0
Group III (n=33)	8	19	3	3

**Table III:** Duration of injury before surgery

	Group I (n=33)	Group II (n=32)	Group III (n=33)
3 weeks to 3 months	16	4	6
3 months to 6 months	4	6	2
6 months to 1 year	2	11	3
1 year to 3 years	7	9	16
3 years to 5 years	7	9	16
> 5 years	1	1	3

In Group I, Group II and Group III mean KT-1000 score was 1.68, 2.26 and 2.24, respectively, mean Lysholm score was 98.30, 97.30 and 97.10, respectively and mean thigh circumference difference was 1.40, 1.90 and 1.60, respectively. Results of KT-1000 score, Lysholm score, Tegner score, difference in thigh circumference and KL grading are shown in Table IV and Table V. Group I was found to be better than Group II and Group III in terms of KT-1000 score. There was no significant difference among the groups when compared in terms of Lysholm score, Tegner score, difference in thigh circumference and KL grading.

**Table IV:** Results in different groups

	Group I (n=33)	Group II (n=32)	Group III (n=33)	P value*
KT-1000 score (Mean +/- S.D)	1.68 +/- 0.27	2.26 +/- 0.19	2.24 +/- 0.22	1.11
Lyshom score (Mean +/- S.D)	98.30 +/- 2.40	97.30 +/- 2.80	97.10 +/- 4.50	0.12
Tegner score				
Pre-injury	8.27 +/- 1.90	8.30 +/- 1.80	8.10 +/- 1.20	0.80
Final follow-up	7.80 +/- 1.70	7.20 +/- 2.60	7.78 +/- 2.20	0.40
Mean difference	0.45	0.39	0.39	
Thigh circumference difference (cm)	1.40 +/- 0.20	1.90 +/- 0.12	1.60 +/- 0.70	3.61
(Mean +/- S.D)				
KL grading				
Grade 0	11	8	7	
Grade 1	22	22	26	
Grade 2	0	2	0	
Grade 3	0	0	0	
Grade 4	0	0	0	

*ANOVA test

**Table V:** Comparison among groups

	KT-1000 (P value*)	Lyshom (P value*)	Tegner (P value*)	TC (P value*)
Group I vs Group II	0.01	0.30	0.42	0.18
Group I vs Group III	0.01	0.12	0.89	0.50
Group II vs Group III	0.89	0.86	0.53	0.10

TC: thigh circumference difference *Post Hoc Tukey HSD

## Discussion

ACL deficient knee leads to significant morbidity in a high demand patients. The purpose of ACL reconstruction is to resume the stability of the knee to pre injury level without significant complications. Despite being debated for many years, there is still controversy about the optimal graft choice for ACL reconstruction. BPTB graft is considered to have lesser laxity, high durability and larger graft diameter as compared to STG graft^8,9^ however it has more donor site morbidity^10,11^. Allograft is reported to have significantly lesser stability than autograft and is considered only for multi ligament injury where autograft is insufficient^12,13^.

Though the mechanical stability of reconstructed ACL is reported to range from 85% - 90% this does not correlate with functional outcome^14^. Loss of proprioception after ACL reconstruction is considered to be one of the reasons for this suboptimal outcome^15-17^. Also, it is reported that the fixation at the tibial side is the weakest point in the whole construct of ACL reconstruction when free graft is used^18,19^. The problem of loss of proprioception and fixation at tibial side can be solved by using STG graft with preserved tibial insertion. When graft is not stripped from its tibial side the nerve supply of the tendon and so its proprioception is not disrupted. However, there is insufficient literature comparing this preserved tibial insertion graft to free graft for ACL reconstruction.

BPTB graft heals more rapidly as compared to STG graft because in case of BPTB graft healing takes place at bone-bone interface whereas in STG graft healing takes place at soft tissue-bone interface^20,21^. If the graft is loaded before sufficient healing there is decrease in tension and loosening of the graft leading to decreased stability. Since the healing is better, there are less chances of graft loosening and knee instability with BPTB graft as compared to STG graft. This has also been reported by previous clinical studies^8,9^. However recent meta-analysis failed to find any significant difference between these two types of grafts^22-24^. Xie *et al* in their meta-analysis of seven studies compared the BPTB graft with four stranded hamstring graft^22^. The authors did not find any statistically significant difference between two groups when compared for knee stability using KT-1000 score and Lachman test. However authors reported that stability was better in BPTB group when assessed by pivot shift test. Li *et al* in their meta-analysis of nine studies (738 patients) found similar results^23^. When compared for knee stability using Lachman test there was no significant difference between BPTB and hamstring graft but BPTB was found to be significantly better than hamstring when compared by pivot shift test. Similar results were found by Magnussen *et al* in their meta-analysis of seven studies when knee stability was compared by using Lachman test^24^. The authors did not find any significant difference between BPTB and hamstring grafts. However, unlike the two above mentioned studies, the authors found that the results were comparable between BPTB and hamstring graft even when compared for pivot shift test. In our study we assessed knee stability on KT-1000 score and found that KT-1000 score is significantly better in BPTB graft as compared to free STG and STGPI grafts. Our study is different than previous studies as we also compared BPTB graft with STGPI graft. We used KT-1000 score to assess knee stability. We believe that KT-1000 score is no less reliable than other tests to assess knee stability. Though it is believed that Lachman test and Pivot test indicates anterior stability and rotational stability respectively, the relationship between Pivot test and stability needs a clarification^23^. Also, Abulhasan *et al* in their systematic review of 34 articles evaluated different knee stability measures and concluded that there is no gold standard technique to assess knee stability^25^. Kilinc *et al* compared the accuracy of the Lachman and Anterior Drawer Tests to evaluate the knee examination with the KT-1000 arthrometer in 40 ACL reconstructed knees^26^. The authors concluded that KT-1000 score is as reliable as Lachman and Anterior Drawer tests.

In our study we analysed the patient reported outcome using Lysholm score. We did not find any statistically significant difference among the three groups. Xie *et al*^22^ in 2015 and Li *et al*^23^ in 2012, in their meta-analysis, analysed the patient reported outcome using IKCD and did not find any difference between BPTB and hamstring grafts after ACL reconstruction. Similarly Magnussen *et al* in 2011 in their meta-analysis analysed patient reported outcome after ACL reconstruction using Lysholm score, IKCD and Cincinnati scores and find no difference between BPTB and hamstring grafts^24^. Though IKDC score is well accepted to provide a full evaluation of the post-operative ACL knee reconstruction outcome, Lysholm score, which we used in our study, is also believed to be an acceptable psychometric parameter as patient-administered score after ACL reconstruction^27^.

Tegner score is a standardised method of grading work and sporting activity. In our study we did not find any statistically significant difference among the groups when assessed for return to sports activity using Tegner score and difference in thigh circumference from normal limb. Our results are different than previously reported results by Magnussen in their meta-analysis^24^. The authors found that return to preinjury activity level is favoured by BPTB group as compared to STG group though the functional outcome was same in both groups.

Associated meniscus injury may lead to degenerative changes after ACL reconstruction. In our study we excluded the patients having associated meniscus injuries. We used KL grading to see osteoarthritic changes after ACL reconstruction and found no significant difference among the tree types of grafts we used.

Similar to the reported results in previous meta-analysis^22-24^ we did not find any difference between graft failure rates of BPTB and free STG group. In addition we also compared the failure rates of STGPI grafts with BPTB and free STG groups and we did not find any difference among the groups.

We acknowledge the drawbacks of our study. Our study is a post-hoc analysis of retrospective cohort and we believe a better designed prospective study with a bigger sample size can be more valuable.

## Conclusion

After ACL reconstruction the BPTB graft is better than free STG and STGPI grafts in terms of knee stability. When compared for patient reported outcome, return to sports activity, osteoarthritic changes and graft failure there is no significant difference among the three types of grafts at mid to long term follow-up. However, we suggest further a better designed study on this topic.
